# Evaluation of Four Rapid Tests for Detection of Hepatitis B Surface Antigen in Ivory Coast

**DOI:** 10.1155/2020/6315718

**Published:** 2020-06-26

**Authors:** Bamory Dembele, Roseline Affi-Aboli, Mathieu Kabran, Daouda Sevede, Vanessa Goha, Aimé Cézaire Adiko, Rodrigue Kouamé, Emile Allah-Kouadio, Andre Inwoley

**Affiliations:** ^1^NBTS (National Blood Transfusion Center), Côte d'Ivoire; ^2^Félix Houphouët-Boigny University, Abidjan, Côte d'Ivoire; ^3^Research and Diagnosis Center for AIDS and other infectious diseases (CeDReS), CHU (University Hospital) of Treichville, Abidjan, Côte d'Ivoire; ^4^Institut Pasteur, Côte d'Ivoire; ^5^National Hepatitis Program, Côte d'Ivoire

## Abstract

**Background:**

Hepatitis B virus (HBV) infection is a leading cause of liver disease worldwide. Hepatitis B surface antigen (HBsAg) rapid diagnostic tests (RDTs) could be an ideal tool for a large-scale HBV screening in settings with high endemicity but limited infrastructure. The aim of this study was to evaluate the diagnosis performance of such RDTs for screening HBV infection in Ivory Coast.

**Methods:**

From September 2018 to January 2019, a cross-sectional phase I evaluation study of RDTs was conducted in three laboratories of Abidjan (CeDReS, CNTS and IPCI), on a panel of 405 whole blood samples and 699 plasmas. Four HBsAg RDTs (Determine™ HBsAg, SD Bioline HBsAg WB®, Standard Q HBsAg® and Vikia HBsAg®) were evaluated. The diagnostic performance (sensitivity and specificity) was calculated in comparison to the reference sequential algorithms of two EIA tests (Dia.Pro HBsAg® one version ULTRA and Monolisa™ HBsAg ULTRA).

**Results:**

The Determine™ HBsAg and Vikia HBsAg® tests performed well, with 100% of sensitivity, specificity both on plasma and on whole blood. For SD Bioline HBsAg WB® and Standard Q HBsAg®, the specificities were 99.8% and the sensitivities 99.3% and 97.1% respectively. Finally, there were a total of 19 false negative results: 3 with SD Bioline HBsAg WB® and 16 with Standard Q HBsAg®.

**Conclusion:**

Determine HBsAg® from Alere and Vikia HBsAg® from Biomérieux are the most suitable RDTs for screening for HBV in Ivory Coast. A phase II evaluation must be initiated.

## 1. Introduction

Hepatitis B virus (HBV) is a serious public health problem worldwide and a major cause of chronic hepatitis, cirrhosis, and hepatocellular carcinoma (HCC). The World Health Organization (WHO) estimated that in 2015, 257 million persons, were living with chronic HBV infection in the world with 890 000 deaths from cirrhosis, and HCC [1]. Most countries in Africa are of higher-intermediate endemicity or highly endemic for HBV with a mean prevalence of 6.1% [1, 2]. In Ivory Coast, a sub-Saharan country, hepatitis B virus surface antigen (HBsAg) prevalence was estimated at 4.3 among schoolchildren [3], 8.48% in the general population [4] and 11% in the blood donors [5].

Early diagnosis is critical in reducing hepatitis-related morbidity and mortality, as well as identifying candidates for HBV vaccination. HBsAg is used as the marker of infection for both HBV screening and to detect suspected acute cases or chronic hepatitis B in any community [6]. HBsAg is typically detected using sensitive immunoassays with an immunoassay analyzer in a hospital laboratory setting. Although such enzyme immunoassays (EIAs) can effectively detect the viral antigens, they have high costs, require dedicated facilities, sophisticated equipment, trained technicians, and a continuous supply of electricity, and have long turnaround times [7]. These drawbacks of laboratory-based immunoassays limit their usefulness in resource-limited settings [8]. For these reasons, WHO recommends the use of inexpensive simple and rapid tests with performance comparable to the immunoassays that can be performed by the laboratories of peripheral health centers in resource-limited countries. Rapid diagnostic tests (RDTs) are quantitative immunochromatographic tests for the purpose of massive screening in non-laboratory environment. It could be designed to detect HBsAg with a very little turnaround time for the test result to be made available for the patients. Several RDTs are circulating and used for hepatitis B screening in Ivory Coast during clinical research and for routine diagnosis without the approval of the Direction of Pharmacy, Drug and Laboratory (DPML) which is the regulatory body of the health ministry of Ivory Coast.

Although some evaluations have been carried out in others countries [9, 10], no evaluation has been made in Ivory Coast as recommended by WHO before the marketing to certify their reliability. This study was carried out to evaluate the performance of four different HBsAg RDTs for marketing in Ivory Coast.

## 2. Materials and Methods

### 2.1. Study Design

From September 2018 to January 2019, a cross-sectional phase I evaluation study of RDTs was conducted at the Institut Pasteur of Ivory Coast (IPCI), the Center for Diagnosis and Research on AIDS and other infectious diseases (CeDRes) and the National Blood Transfusion Center (NBTC). It was initiated by the Direction of Pharmacy, Drug and Laboratory (DPML) in association with the National Program of fight against the viral hepatitis according to the procedures used at national level. The selection criteria of tests were : colorimetric reading between one and two steps; performing tests on serum / plasma and whole blood obtained by venous or capillary sampling; sensitivity and specificity known in other regions of the world on plasma / serum / whole blood; test life greater than 12 months; storage and stability at room temperature (20-30°C).

### 2.2. Assessment Panel

This study was carried out on serum / plasma and whole blood samples. According to WHO RDTs evaluation guide [11], six hundred ninety-nine (699) serum and plasma samples came from the biobank of IPCI and CeDRes, while four hundred and five (405) whole blood samples were collected from blood donors at the National Blood Transfusion Center. Approximately, 4 ml of whole blood was collected into a tube containing an anticoagulant (heparin or ethylene diaminetetracetic) in addition to the usual tubes provided for the biological qualification of donations. Part of the blood collected was centrifuged to carry out the reference tests. The status (positive or negative) of all specimens was established according to the algorithms used in each laboratory. Briefly, laboratories performed Enzyme Immunoassay (EIA) and or Chemiluminescence immunoassay (CLIA).

### 2.3. Rapid Tests Evaluated

We evaluated four HBsAg RDTs: DetermineTM HBsAg (Alere International Limited, Ballybrit Galway, Ireland), SD Bioline HBsAg WB® (Standard Diagnostics Inc, Korea), Standard Q HBsAg® (SD Biosensor, India) and Vikia HBsAg® (Biomérieux, Marcy l'étoile, France). These qualitative tests are based on immunochromatographic techniques for lateral association of monoclonal and polyclonal antibodies specific for HBsAg. We performed the test according to manufacturer's procedure. All these tests give visual readout.

### 2.4. References Tests

HBsAg status of the panel serum/plasma samples has been determined using a sequential algorithm consisted of two commercially available enzyme-linked immuno-assay (ELISA) tests: Dia.Pro HBsAg® one version ULTRA (Diagnostic BioProbes Srl, Italy) and Monolisa™ HBsAg ULTRA (BIO-RAD, Marnes-la-coquette, France). The result unit used is a ratio of the sample optical density (*OD*) to the threshold value (*TV*). Samples with *OD*/*TV* values higher than or equal to 1.00 are considered reactive. Positive results obtained with Dia.Pro Ag were confirmed with the Monolisa AgHBs ULTRA®.

### 2.5. Statistical Analysis

The sensitivity, specificity, positive predictive value (PPV),negative predictive value (NPV) and accuracy of these four kits were calculated with 95% confidence interval according to the existing formula [12]. Kappa concordance value was also calculated and interpreted according to the criteria proposed by Landis and Koch [13]. The diagnostic performances were compared between the plasma/serum and whole blood samples and statistical significance of differences in diagnostic performances were determined using Fisher's exact test. P ≤ 0.05 were considered statistically significant.

## 3. Results

### 3.1. Diagnostic Performance and Accuracy

The performance of each of the four RDTs evaluated in this study is shown in Table 1. The Determine™ HBsAg and Vikia HBsAg® tests have shown better performance, with 100% of a sensitivity, specificity, PPV, NPV and an accuracy rate. However, all the four tests showed a good agreement (k>0.97). In both plasma/serum and whole blood samples, the performances of each of the four commercially available rapid kits were comparable (Table 2).

### 3.2. Discordance Results

Globally, there were a total of 19 false negative results: 3 with SD Bioline HBsAg WB® and 16 with Standard Q HBsAg® for a discordance rate of 0.36% and 1.54% respectively. Details are resumed in Table 3.

## 4. Discussion

Many HBsAg rapid tests using immunochromatographic assays have been commercialized worldwide. The major challenge for these tests is to detect the low levels of the target antigen that are present in a relatively high proportion of asymptomatic carriers [14, 15]. Therefore, in a poor resource setting where EIA is unavailable, rapid diagnosis test is a reasonable alternative for epidemiologic surveys due to the lower cost of testing and simpler logistics [8]. As recommended by WHO, in the present study, we evaluated the performance of four HBsAg RDTs.

We found that Determine™ HBsAg and Vikia HBsAg® tests had best technical performance. Global specificities obtained with Determine™ HBsAg and Vikia HBsAg® were similar to those reported by previous studies (100%) [9, 10, 16]. By contrast, sensitivities were slightly higher than those reported in previous evaluations between 82.5% and 98% [9, 16, 17].

Many studies revealed that the sensitivity and specificity of the SD Bioline WB kits were 94.1 to 100% and 99.3 to 100% for the HBsAg kit [18, 19], values which were similar to our findings. However, Farooq et al. have found low sensitivity (17,4%) with SD Bioline HBsAg WB in healthy blood donors of Pakistan using PCR as gold standard [20].

The disparity of results on the sensitivity and specificity of rapid tests could be explained by the existence of mutant viruses which have modified surface antigens (HBsAg), thus making their detection by routine immunological techniques impossible [20, 21]. There are many HBsAg-immune-escape mutants those can be found both within and without the “a” determinant. The first mutant described, and which remains the most prevalent is G145R. Indeed, Studies reported HBs Ag mutant prevalence ranging from0,7% to 14,8% depending on the populations studied [22–25].

Also lower HBsAg concentration and viral load could lead to a false negative reaction [9, 20, 21]. Antibodies included in reagents do not take into account all mutated antigens.

Regarding the Standard Q HBsAg, no previous study, apart from the one reported by the manufacturer, has been documented, not allowing us to make comparison.

According to current WHO procurement eligibility for HBsAg assays that require that RDTs assays might have a diagnostic sensitivity and specificity of > 99% and > 98% respectively [26], three of the four evaluated tests (Determine TM HBsAg, Vikia HBsAg® and SD Bioline HBsAg WB®) can be accepted as HBsAg in vitro Diagnostic Tests.

We did not observe any significant difference in the performance of the tests evaluated with whole blood and serum/plasma. Njai et al. reported higher sensitivity in serum (88.5% vs 95.3%) but higher specificity in whole blood (100% vs 93.3%) [10]. This difference could be explained by the fact that the dried blood spots (DBS) has been used as a medium for the reference standard instead of serum or plasma.

An important consideration of definitive laboratory diagnosis also relates to controlling for false-positive and false negative results. We noticed a low rate of false negative results only with SD Bioline WB® (0.5%) and Standard Q® (2.8%) in this study. On the contrary, other studies have reported false-negative results of Determine and Vikia HBsAg tests. They associated this with a low HBsAg concentration, HBsAg mutants, low viral load, and certain viral genotypes [9, 10, 21]. Moreover false-negative results have a threat of silent transmission and spreading of diseases among people and also create more interest for sensitive assays like EIA.

In our study, the inter-reader variability and variability of results over time were not investigated. These parameters would have allowed us to appreciate the stability of the test results over time.

## 5. Conclusion

Among the four RDTs evaluated in this study, Determine™ HBsAg and Vikia HBsAg® tests should be appropriate for HBV screening and marketed in Ivory Coast. These two tests are rapid, simple and very suitable for peripheral laboratories and provided excellent performances both on serum / plasma and on whole blood. As recommended by the World Health Organization, Ivory Coast must now start the phase II of evaluation of these two RDTs to validate these performances under field conditions in an environment characterized by limited resources and multiple public health priorities.

## Figures and Tables

**Table 1:** Performances of Determine™, SD Bioline WB®, Standard Q® and Vikia® for HBsAg detection compared with EIA tests.

	HBsAgserologyEIA		Sensitivity, % (95% CI)	Specificity, % (95% CI)	PPV, % (95% CI)	NPV, % (95% CI)	Accuracy, % (95% CI)	Kappa (95% CI)
	Positive	Négative						
Determine™								
Positive	551	0	100	100	100	100	100	1
Negative	0	553	(99.17-100)	(99.17-100)	(99.17-100)	(99.17-100)	(99.17-100)	
SD Bioline WB®								
Positive	548	1	99.46	99.82	99.82	99.46	99.64	0.99
Negative	3	552	(98.33-99.89)	(98.88-100)	(98.87-100)	(98.34-99.89)	(99.04-99.89)	(0.98-1)
Standard Q®								
Positive	535	1	97.1	99.82	99.81	97.18	98.46	0.97
Negative	16	552	95.3-98.24	98.88-100	98.84-100	95.44-98.29	97.53-99.06	0.95-0.98
Vikia®								
Positive	551	0	100	100	100	100	100	1
Negative	0	553	99.17-100	99.17-100	99.17-100	99.17-100	99.17-100	

Abbreviations: PPV, positive predictive value, NPV, negative predictive value, CI, confidence interval.

**Table 2:** Comparison of Diagnostic accuracy between the plasma/serum and whole of Determine™, SD Bioline WB®, Standard Q® and Vikia® for HBsAg detection.

	Sensitivity, % (95% CI)	Specificity, % (95% CI)	PPV, % (95% CI)	NPV,% (95% CI)	Accuracy, % (95% CI)	P∗
Determine™						
plasma/serum	100 (99.17-100)	100 (99.17-100)	100 (99.17-100)	100 (99.17-100)	100 (99.17-100)	0,91
Wholeblood	100 (99.17-100)	100 (99.17-100)	100 (99.17-100)	100 (99.17-100)	100 (99.17-100)	
SD Bioline WB®						
plasma/serum	98.84 (95.61-99.95)	99.82 (98.88-100)	99.82 (98.87-100)	98.93 (95.93-99.96)	99.64 (99.04-99.89)	0,38
Wholeblood	100 (99.17-100)	100 (99.17-100)	100 (99.17-100)	100 (99.17-100)	100 (99.17-100)	
Standard Q®						
plasma/serum	96.53 (92.47-98.57)	99.82 (98.88-100)	99.81 (98.84-100)	97.18 (95.44-98.29)	98.46 (97.53-99.06)	0,42
Wholeblood	98.05 (94.91-99.42)	100 (99.17-100)	100 (99.17-100)	98.04 (94.89-99.41)	99.01 (97.33-99.71)	
Vikia®						
plasma/serum	100 (99.17-100)	100 (99.17-100)	100 (99.17-100)	100 (99.17-100)	100 (99.17-100)	0,91
Wholeblood	100 (99.17-100)	100 (99.17-100)	100 (99.17-100)	100 (99.17-100)	100 (99.17-100)	

Abbreviations: PPV, positive predictive value, NPV, negative predictive value, CI, confidence interval. ^∗^Calculated by Fisher's test.

**Table 3:** Results observed with the Monolisa test for samples giving false results with any of the rapid diagnosis tests.

Identity	OD/TV^a^ with Monolisa AgHBs Ultra®	Status HBsAg^b^	Results of rapid diagnosis tests			
			Determine™ HBsAg	SD Bioline HBsAg WB®	Standard Q HBsAg®	Vikia HBsAg®
7402926	33,7	Positive	Positive	Positive	Negative	Positive
9449856	43,8	Positive	Positive	Positive	Negative	Positive
1457809	32,0	Positive	Positive	Positive	Negative	Positive
9441806	41,2	Positive	Positive	Positive	Negative	Positive
ETR 036B/18	31,9	Positive	Positive	Positive	Negative	Positive
ETR 037B/18	33,1	Positive	Positive	Negative	Negative	Positive
ETR 045B/18	34,0	Positive	Positive	Positive	Negative	Positive
ETR 048B/18	34,6	Positive	Positive	Positive	Negative	Positive
ETR 052B/18	27,9	Positive	Positive	Positive	Negative	Positive
ETR 164B/18	21,1	Positive	Positive	Positive	Negative	Positive
C-019	32.3	Positive	Positive	Positive	Negative	Positive
C-345	32.3	Positive	Positive	Negative	Negative	Positive
S-262	29.5	Positive	Positive	Positive	Positive	Positive
S-169	23.6	Positive	Positive	Positive	Negative	Positive
YOP HGE 022	32.9	Positive	Positive	Negative	Negative	Positive
H-3569	32.3	Positive	Positive	Positive	Negative	Positive
HGT HGE 011	12,5	Positive	Positive	Positive	Negative	Positive
ETR 144B/18	1,3	Negative	Negative	Positive	Positive	Negative

^a^Ratio of the sample optical density (*OD*) to the threshold value (*TV*), as calculated by Monolisa AgHBs Ultra®. Samples with *OD*/TV values greater than or equal to 1.00 are considered reactive. ^b^ Determined with two EIA tests: Dia.Pro HBsAg® one version ULTRA for detection of HBsAg; positive samples were confirmed using Monolisa™ HBsAg ULTRA Confirmation test.

## Data Availability

The data used to support the findings of this study are available from the corresponding author upon request.
